# Third-Generation Antipsychotics: The Quest for the Key to Neurotrophism

**DOI:** 10.3390/life15030391

**Published:** 2025-03-01

**Authors:** Federico Mucci, Alessandro Arone, Riccardo Gurrieri, Francesco Weiss, Gerardo Russomanno, Donatella Marazziti

**Affiliations:** 1 Department of Psychiatry, Lucca Zone, Azienda USL Toscana Nord Ovest, 55100 Lucca, Italy; federicomucci@gmail.com; 2 Department of Clinical and Experimental Medicine, Section of Psychiatry, University of Pisa, 56100 Pisa, Italy; alessandroarone2@gmail.com (A.A.); riccardogurrieri4@gmail.com (R.G.); francesco.weiss93@gmail.com (F.W.); g.russomanno93@gmail.com (G.R.)

**Keywords:** neuroplasticity, neurotrophism, third-generation antipsychotics, brexpiprazole, cariprazine, lurasidone, pimavanserin, roluperidone, schizophrenia, cognitive enhancement

## Abstract

Antipsychotic drugs (APs) have profoundly changed the treatment landscape for psychiatric disorders, yet their impact on neuroplasticity and neurotrophism remains only partially understood. While second-generation antipsychotics (SGAs) are associated with a better side effect profile than their predecessors, the emergence of third-generation antipsychotics (TGAs)—such as brexpiprazole, cariprazine, lurasidone, iloperidone, lumateperone, pimavanserin, and roluperidone—has prompted renewed interest in their potential neuroprotective and pro-cognitive effects. This review attempts to carefully examine the evidence on the neurotrophic properties of TGAs and their role in modulating brain plasticity by analyzing studies published between 2010 and 2024. Although data remain limited and focused primarily on earlier SGAs, emerging findings suggest that some TGAs may exert positive effects on neuroplastic processes, including the modulation of brain-derived neurotrophic factors (BDNFs) and synaptic architecture. However, robust clinical data on their long-term effects and comparative efficacy are lacking; therefore, further research is necessary to validate their role in preventing neurodegenerative changes and improving cognitive outcomes in patients with psychiatric conditions.

## 1. Introduction

Since the serendipitous discovery in the mid-1950s and rapid introduction into the clinical practice of chlorpromazine, the first antipsychotic drug (AP), the paradigms of psychiatric care have dramatically shifted toward the pharmacological management of previously untreatable disorders [1]. Nobel Prize winner Arvid Carlsson and colleagues later discovered that the antagonism of dopamine type-2 (D2) receptors underlies the antipsychotic effect and that psychosis is often associated with altered dopaminergic activity [2]. Despite subsequent advances in our understanding of psychosis, the D2 receptor blockade remains central to the mechanism of action of antipsychotic drugs [3].

Traditionally, antipsychotics are categorized into typical or first-generation antipsychotics (FGAs) and atypical or second-generation antipsychotics (SGAs) [4]. FGAs differ mainly in their potency, linked to varying affinities for D2 receptors [4]. SGAs, by contrast, were introduced with the goal of maintaining antipsychotic efficacy while reducing the incidence of extrapyramidal symptoms (EPSs) and hyperprolactinemia that characterize FGAs [5]. In practice, SGAs interact with multiple receptors, but can lead to significant metabolic side effects such as weight gain, dyslipidemia, insulin resistance, and increased cardiovascular risk [6]. Over time, SGAs have been approved for several psychiatric conditions, including treatment-resistant schizophrenia, bipolar disorder, schizoaffective disorder, and as adjunctive treatments for major depressive disorder (MDD) [7]. Their widespread clinical use thus extends well beyond the initial indication of psychosis.

Although the pathophysiology of psychiatric disorders is still incompletely understood, it is currently seen as the product of multiple interactions between genetic vulnerability, environmental factors (e.g., life events), and internal biological processes (including neurotransmission, neurotrophism, inflammation, and immune activity) [8,9,10]. Early psychopharmacological research used drugs such as antipsychotics and other psychotropics as primary research tools to investigate these biological underpinnings [11]. With the emergence of more advanced techniques, a growing body of neuroimaging studies has revealed a variety of structural and functional brain changes in disorders like schizophrenia—such as reductions in brain volume and white/gray matter—and in MDD, including smaller hippocampal and anterior cingulate volumes, amygdala alterations, and cortical thinning [12,13,14,15,16,17]. Over the years, evidence has also accumulated regarding the long-term effects on the brain of frequently used psychiatric medications, such as antidepressants [18,19] and mood stabilizers [20], which are often regarded as displaying neuroprotective properties. However, data on antipsychotics remain more controversial, even though neuroplasticity and neurogenesis are hypothesized to play significant roles in the pathophysiology of schizophrenia.

In the past three decades, case reports and clinical studies have progressively documented neurotoxic effects possibly linked to FGAs while suggesting that SGAs may instead exert neuroprotective influences [21,22]. Several investigations have focused on identifying secondary neurotrophic and neuroprotective actions that could contribute to the therapeutic profile of SGAs [21,22,23,24,25]. While encouraging, most of the available data concern older SGAs. More recent drug development appears to be moving toward pro-cognitive and neurotrophic efficacy [25]. Consequently, there is still uncertainty regarding whether novel antipsychotic compounds—sometimes referred to as third-generation antipsychotics (TGAs)—offer distinct or superior pro-cognitive and neurotrophic benefits compared to conventional SGAs [23,24]. TGAs of interest include brexpiprazole, cariprazine, lurasidone, iloperidone, lumateperone, pimavanserin, and roluperidone, which are already in clinical use primarily for their antipsychotic effects. The delineation between SGAs and TGAs may not be merely nominal but could reflect meaningful differences in pharmacodynamic profiles—particularly partial agonism at dopamine receptors—and translate into new therapeutic advantages. A deeper understanding of these novel mechanisms could be crucial for refining treatment strategies and fostering the development of antipsychotics with improved efficacy and tolerability.

In light of the growing emphasis on preventing neurodegenerative complications in psychiatric and neurological conditions, the present review aims to provide a balanced overview of the limited yet emerging evidence regarding the potential neuroprotective and neurotrophic properties of these newer antipsychotic agents, presenting its results accordingly to the PRISMA extension for Scoping Reviews (PRISMA-ScR) [26]. The ultimate goal is to outline whether these agents might soon become standard tools in clinical practice not only for managing psychosis but also for mitigating long-term neurocognitive decline.

Before proceeding with this review, it appears useful to delineate key terminologies to ensure clarity and precision. The term neuroplasticity refers to the brain’s intrinsic capacity to undergo structural and functional modifications in response to intrinsic or extrinsic stimuli. This encompasses alterations in synaptic strength, dendritic branching, and even the genesis of new neurons, facilitating learning, memory consolidation, and recovery from neural injuries. Such plastic changes are fundamental to the brain’s adaptability and resilience. In parallel, neurotrophism pertains to the mechanisms that support the growth, survival, and differentiation of neurons. This involves the action of neurotrophic factors—proteins that play a pivotal role in neuronal development, maintenance, and repair [27,28]. Given their potential modulation by second- and third-generation antipsychotics, understanding their role provides a necessary framework for the discussion that follows.

### Neuroplasticity and Neurotrophic Factors

Different types of molecules, such as neurotrophins or neurotrophic factors, and cellular adaptations are involved, and neuroplasticity mainly includes biological mechanisms related to synaptic transmission, protein kinases, gene expression and protein synthesis [29]. Neurotrophic factors belong to the class of growth factors (GFs), a group of proteins that play a key role in several cellular processes, such as proliferation, differentiation, maturation, the survival of neurons, and plasticity [30]. They include three main different families of GFs: neurotrophins, glial cell line-derived neurotrophic factor (GDNF) family ligands, and the so-called cytokine family, a heterogeneous group of different molecules [31,32]. These three have been associated with reduced synaptic connectivity in animal models [33]. In mammals, structurally similar proteins, the so-called brain-derived neurotrophic factor (BDNF), the nerve growth factor (NGF), neurotrophin 3 (NT-3), and neurotrophin 4 (NT-4) are the four major neurotrophins [34]. Their biological effects are induced by binding to one or more of the three tyrosine kinase receptors: tropomyosin receptor kinase A, -B, -C (TrkA, TrkB, TrkC) [35]. They also bind, although less specifically, to the p75 neurotrophin receptor (p75NTR) [36]. Neurotrophins seem to be implicated in a wide range of neuropsychiatric disorders, in particular, MDD [37], bipolar disorder [38] and schizophrenia [39], as well as neurodegenerative conditions, such as Parkinson’s disease [40]. The current clinical evidence is stronger for BDNFs and NGFs that represent the most studied neurotrophins so far [41,42]. Regarding psychiatric disorders, a correlation between BDNF low serum levels and schizophrenia was also demonstrated, up to the point that, albeit nonspecific, this abnormality has been considered a cue of involvement of neurotrophic processes in schizophrenia [43,44,45,46]. Interestingly, different observations highlighted that the interaction with neurotrophic factors seems to be more robust for SGAs than for FGAs, as the first may modify BDNF levels [47]. In addition, different compounds would modulate BDNFs in a substantially heterogeneous way. This is supported by the result of a study using longitudinal magnetic resonance imaging that showed how higher levels of BDNFs in schizophrenic patients undergoing SGAs was paralleled by a decline in the progressive loss of cortical gray matter, particularly in the temporal lobe [41,48]. Nevertheless, the relationship between APs and the BDNF needs to be yet clarified. If it is true that some studies detected differences in serum BDNF levels following treatment with SGAs versus FGAs [49,50], it is equally true that other studies were unable to replicate these findings, as no differences were even found with treatment with any kind of APs [51,52]. The nerve growth factor (NGF) is the first member of the neurotrophin family to be characterized [53]. Its main functions include the regulation of growth, differentiation, regeneration, development, and maintenance of neurons even in the peripheral nervous system, and it is also strictly related to the function of cholinergic neurons in the CNS [54,55]. The blood levels of NGFs have been reported to be lower amongst first-episode schizophrenic patients, as compared with healthy subjects [56] but also in depressed [57,58] or bipolar patients [59]. There is some uncertainty regarding the effects of APs on NGF levels, although some data, still a matter of debate, suggest that APs, but especially SGAs, may promote neuronal plasticity [60,61,62,63,64,65,66]. Indeed, it has been shown in rats that olanzapine, an SGA, may increase NGF levels in the hypothalamus, hippocampus, and the occipital cortex [41]. Furthermore, an increase in NGF-induced neuritis outgrowth has been demonstrated with other SGAs, specifically, quetiapine and clozapine [60]. As underlined in a recent review [67], the neurodevelopmental hypothesis of schizophrenia is increasingly supported, as intriguing data highlight synaptic plasticity alterations and disruptions in postsynaptic density (PSD) within patients experiencing psychosis relative to healthy controls [68,69].

First-generation antipsychotics such as haloperidol predominantly modulate subcortical regions, notably the striatum, and these effects have been linked to elevations in immediate early gene (IEG) expression after acute as well as prolonged administration [70,71,72]. Nonetheless, the modulation of cortical activity is less consistent, with evidence of both up- and downregulation of IEG levels [73,74]. By contrast, SGAs, including quetiapine, olanzapine, and clozapine, seem to effectively influence cortical gene expression—specifically increasing BDNF levels in the hippocampus and prefrontal cortex—while also impacting subcortical structures such as dorsal and ventral striatal areas [75,76,77,78,79,80,81]. Clozapine, which remains the sole medication officially indicated for treatment-resistant schizophrenia [82], has been demonstrated to refine cortical functional connectivity and neuronal synchrony in high-gamma frequencies. Moreover, it restores coherence between the prefrontal cortex (PFC) and the medial ventral striatum in preclinical models that have been disrupted by NMDA receptor antagonists like phencyclidine (PCP) and ketamine, a process mediated by its interaction with 5-HT receptors [83,84,85,86].

In drug-naïve first-episode psychosis patients, SGA monotherapy (e.g., risperidone) has been associated with a marked increase in functional connectivity between the cingulate cortex and the PFC, as measured through resting-state fMRI, accompanied by improvements in positive symptomatology [87]. Additionally, clozapine’s therapeutic efficacy correlates with alterations in connectivity patterns between the caudate nucleus and frontal and parietal regions, occurring in the absence of significant shifts in striatal dopaminergic function [88].

## 2. Materials and Methods

We conducted a scoping review to map the existing evidence on the potential neurotrophic effects of recently introduced antipsychotics sometimes referred to as “third-generation antipsychotics” (TGAs). We followed the guidelines outlined by the PRISMA-ScR [26] to ensure transparency in our search and selection processes.

### 2.1. Objectives

The primary aim of this scoping review was to achieve the following:

- Identify and catalog the available literature (both clinical and preclinical) examining possible neurotrophic or neuroprotective effects of TGAs.
- Describe the nature, scope, and methods of these studies.
- Summarize the main findings and highlight gaps for future research.

### 2.2. Search Strategy

A comprehensive literature search was performed across five electronic databases: PubMed, Scopus, Embase, PsycINFO, and Google Scholar. Articles published from 1 January 2010 to 31 August 2024 were considered. We combined relevant MeSH (where applicable) and free-text terms related to neurotrophic factors, neuroplasticity, and specific antipsychotic agents introduced in the last decade, as follows:

(“Neuroplasticity” OR “Neurotrophism”) AND (“Cariprazine” OR “Brexpiprazole” OR “Lurasidone” OR “Iloperidone” OR “Lumateperone” OR “Pimavanserin” OR “Roluperidone” OR “third-generation antipsychotics”)

We also manually searched reference lists of relevant articles and recent reviews to identify additional records that might have been missed during the primary search. No attempt was made to locate the unpublished or gray literature beyond what was indexed in the above databases.

### 2.3. Eligibility Criteria

We included studies if they performed the following:

▪ Investigated one or more of the following compounds in humans or animal models: brexpiprazole, cariprazine, lurasidone, iloperidone, lumateperone, pimavanserin, or roluperidone.
▪ Reported any neurotrophic, neuroprotective, or neuroplastic outcome (e.g., changes in the BDNF, NGF, GDNF, synaptic markers, or relevant imaging findings).
▪ Examined either preclinical (animal or in vitro) or clinical (human) contexts, including case studies, conference abstracts, or short communications published in peer-reviewed or indexed journals.
▪ Were written in English.

Exclusion criteria were as follows:

▪ Studies not focusing on the neurotrophic or neuroprotective outcomes of interest.
▪ Studies that did not involve original data (e.g., expert opinion pieces, editorials, or purely theoretical articles) unless they presented secondary analyses or re-analyses with specific neurotrophic data.
▪ Publications not in English.

### 2.4. Study Selection

All retrieved citations were exported to a reference manager (EndNote). Titles and abstracts were preliminarily screened against the eligibility criteria. Full-text articles were retrieved if deemed potentially relevant or if eligibility remained unclear. Any discordances were resolved through discussion amongst the authors.

### 2.5. Data Charting Process

To ensure consistency, we developed a standardized charting form to extract relevant information from each included study. The variables included the following:

▪ Study design (preclinical vs. clinical, observational vs. experimental).
▪ Sample (e.g., animal species, cell line, patient characteristics).
▪ Intervention (specific TGA, dose, duration).
▪ Neurotrophic/neuroplastic outcomes (e.g., BDNF levels, neuroimaging findings, gene expression profiles).
▪ Main results (summary of neuroprotective or neurotrophic effects).
▪ Conclusions (authors’ interpretation, study limitations).

### 2.6. Synthesis of Results

▪ Given the exploratory nature of a scoping review and the heterogeneity of the included studies, no meta-analysis or formal assessment of bias was undertaken. Instead, we conducted the following:
▪ Summarized key findings qualitatively by organizing them according to the specific antipsychotic agent studied.
▪ Where relevant, subdivided each agent’s evidence into preclinical and clinical investigations to facilitate comparison.
▪ Highlighted methodological gaps or limitations in the existing research.

**Figure 1:**
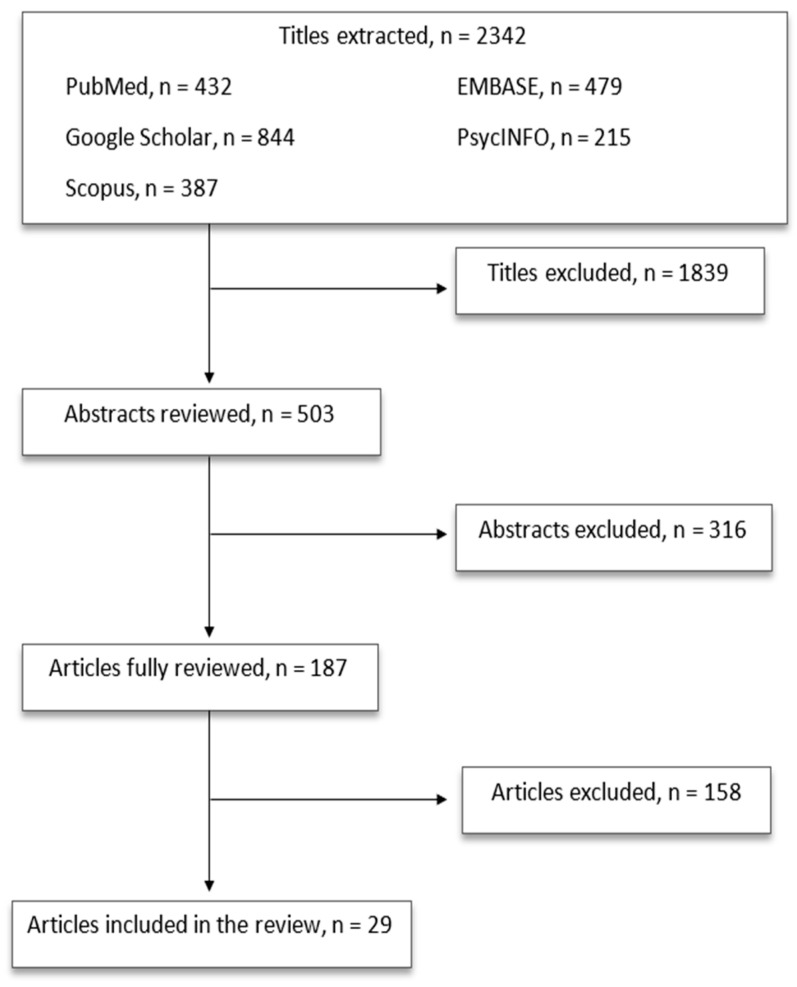
Article selection flow chart.

A modified PRISMA-ScR flow diagram detailing the number of records identified, screened, and included is provided in Figure 1.

## 3. Results

**Figure 2:**
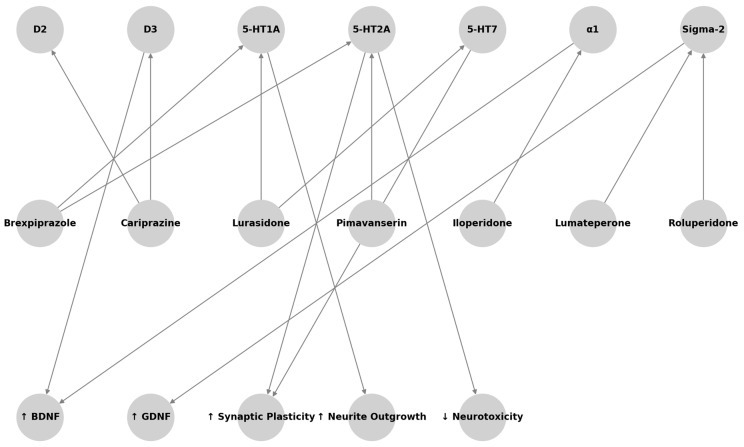
Hypothesized pathways by which TGAs may confer neurotrophic or neuroprotective benefits. Receptor interactions include partial agonism at dopamine D2/D3 receptors (cariprazine, brexpiprazole), antagonism at serotonin 5-HT2A receptors (pimavanserin, lumateperone), and others. These modulations can trigger downstream signaling cascades (e.g., involving BDNF, GDNF, mTOR), leading to enhanced neuronal survival, synaptic plasticity, and potentially improved cognitive outcomes.

The first selection excluded 1839 titles because of the following characteristics: (a) duplicates; (b) not concerning the scope of this paper; and (c) not informative enough. The second selection excluded 316 abstracts after being read and reviewed, as the information reported did not fulfill the scope of our paper and/or the presented information did not seem relevant to the discussed topic. Subsequently, 158 articles were excluded after being completely read and evaluated, as they did not provide enough information and/or did not result sufficiently in line with our review. Finally, 29 papers were included in the present review (Figure 1). In the next chapters, the main characteristics of the latest neuroleptic compounds with the available information on their neurotrophic activity, if any, will be reviewed (Figure 2) (Table 1, Table 2 and Table 3).

### 3.1. Brexpiprazole

#### 3.1.1. Pharmacological Profile and Clinical Use

Brexpiprazole is one of the latest antipsychotics introduced into clinical practice. Its pharmacological profile is similar, although not identical, to that of aripiprazole. It acts as a serotonin-dopamine activity modulator by displaying potent antagonism at 5-HT2A receptors, as well as at α1b/2c adrenoreceptors, and partial agonism at 5-HT1A and D2 receptors. However, unlike aripiprazole, brexpiprazole exhibits reduced intrinsic activity on D2 receptors, a more potent antagonism at 5-HT2A, and a greater binding affinity for the norepinephrine transporter (NET). This would lead to a reduced incidence of the typical effects mediated by partial agonism on D2 receptors, i.e., restlessness, akathisia, nausea, and insomnia. Brexpiprazole also shows a moderate affinity for histamine receptors H1, an important feature that explains a reduced intensity of adverse events such as weight gain and sedation [89,90,91]. Brexpiprazole is mainly used for the treatment of schizophrenia and as an add-on treatment for MDD [92,93,94]. Regarding schizophrenia, both its efficacy and tolerability in acute treatment [95,96,97] and in relapse prevention [98] were demonstrated. Moreover, a possible role of this drug in the treatment of bipolar depression is also emerging, since it could reduce both the extent of depressive symptoms and improve quality of life [99].

#### 3.1.2. Preclinical (Animal/Translational) Evidence

A series of data demonstrates that brexpiprazole may also potentiate neurotrophic processes, as it can enhance neurite outgrowth induced by NGFs in P12 cells through binding to 5-HT1A and 5-HT2A receptors [100]. Indeed, P12 cells represent a cell line derived from a pheochromocytoma of the rat adrenal medulla that has long been used as a model system for NGF-induced outgrowth [101,102]. In addition to the aforementioned serotonergic receptor subtypes, it has also been demonstrated that brexpiprazole-induced neurite overgrowth might involve Ca^2+^ signaling via inositol 1,4,5-triphosphate (IP3) receptors [100], and even the essential molecular chaperone Hsp90, which contributes to the assembly of several protein complexes [102,103]. Moreover, a combination of brexpiprazole and fluoxetine, when compared to the separate use of the two drugs, showed encouraging antidepressant results in a mouse model of inflammation-induced depression (following lipopolysaccharide, LPS, administration). The antidepressant effect of this combination was supported by findings such as a significant attenuation of immobility time and decreased BDNF levels in different brain areas (prefrontal cortex, CA1 of the hippocampus, and dentate gyrus [DG]), as well as an attenuation of the LPS-induced decrease in spine density in the ventral region of the medial prefrontal cortex, CA3 of the hippocampus, and DG [104]. The authors also demonstrated improved alterations in BDNF-TrkB signaling and dendritic spine density in the prefrontal cortex, hippocampus, and nucleus accumbens following social defeat stress in mice [105].

In conclusion, preclinical and limited data would indicate that brexpiprazole might interact with neurotrophic processes.

### 3.2. Cariprazine

#### 3.2.1. Pharmacological Profile and Clinical Use

Cariprazine is an antipsychotic with a unique binding profile [25]. It acts as a partial agonist of D2 and D3 receptors; in particular, it has a greater affinity for D3 receptors compared to the marked D2 antagonism of most previously introduced APs [106,107]. Cariprazine also binds to 5-HT1A receptors with partial agonist activity and acts as an antagonist at 5-HT2B receptors. It shows low affinity for 5-HT2C, H1, and adrenergic receptors, which may partly explain the reduced incidence of adverse events classically associated with antipsychotics [108]. The FDA-approved cariprazine for the treatment of schizophrenia, bipolar disorder type 1 (acute, mixed, or depressive episodes), and new evidence is emerging about its potential role in treating MDD [109,110]. In particular, cariprazine has been shown to reduce anhedonia-like behavior in a chronic mild stress model in rats, suggesting an antidepressant activity [111], and it exhibits a protective effect on cognitive functions, likely due in part to its partial agonism at D3 receptors [112].

#### 3.2.2. Animal/Translational Studies on Neurotrophic Effects

It has been hypothesized that enhanced D3 activity underlies the neuroadaptive changes responsible for cariprazine’s antidepressant activity [113]. Furthermore, a peculiar feature of this drug—unlike other APs—seems to be its ability to increase D3 receptor levels in brain regions rich in these receptors following prolonged treatment. Indeed, cariprazine can occupy D3 receptors even at low doses, causing a major upregulation of D3 receptors. This effect has been linked to the BDNF, which has long been considered to control the development and expression of D3 receptors [114,115,116,117].

In any case, albeit intriguing, these preliminary observations on possible neurotrophic effects of cariprazine are required to be substantiated by further studies.

### 3.3. Lurasidone

#### 3.3.1. Pharmacological Profile and Usage

Lurasidone is an antipsychotic chemically belonging to the benzisothiazole class and structurally related to ziprasidone and perispirone [118]. First approved in 2010 for the treatment of schizophrenia, lurasidone has subsequently been employed in the management of bipolar depression [119,120]. Its main action is full antagonism at D2 and 5-HT2A receptors [121]. Furthermore, lurasidone displays marked binding to 5-HT7 receptors, a moderate affinity as an antagonist for α2C receptors, partial agonism at 5-HT1A receptors, and low affinity for muscarinic M1, histamine H1, α1A, and α2A receptors [122]. This distinctive binding profile has been theoretically associated with the pro-cognitive effects of lurasidone, potentially supporting learning and memory processes via 5-HT7, α2C, and 5-HT1A receptors [121,122,123]. Additionally, its low affinity for H1 and M1 receptors confers a lower risk of sedation and weight gain compared to other SGAs [124], although the same feature can increase the risk of EPS [125].

#### 3.3.2. Preclinical Evidence

It is worth noting that the literature reports more data on the neurotrophic effects of lurasidone than on any other SGA. In rat models of response to acute stress, the modulation of BDNF expression in the prefrontal cortex and hippocampus was examined following chronic treatment with lurasidone [126]. The study found that acute stress induced an increase in BDNF mRNA levels primarily in the prefrontal cortex, with a lesser effect in the hippocampus. Furthermore, rats treated with lurasidone showed an enhanced modulation of BDNF mRNA levels mostly in the hippocampus and, to a lesser extent, in the prefrontal cortex. The authors also reported a parallel increase in mature BDNF protein levels in the prefrontal cortex, suggesting a possible neuroprotective action relevant to cognitive deficits in schizophrenia [126]. In other studies, lurasidone’s neurotrophic role was supported by findings in additional experimental contexts. A combination of lurasidone with valproate stimulated neuroadaptive changes by increasing BDNF expression in the ventral hippocampus through the regulation of specific neurotrophin transcripts [127]. Also, lurasidone was shown to modulate synaptic and neuroplastic proteins in an experimental model of depression, potentially restoring BDNF mRNA in the prefrontal cortex and normalizing levels of glial fibrillary acidic protein (Gfap), postsynaptic density protein 95 (Psd95), and certain regulators of protein translation at the synapse, such as mTOR and eukaryotic elongation factor 2 (EEF2) [128]. Since Gfap and PSD-95 levels are often reduced in depression [129,130], and mTOR/EEF2 pathways are implicated in psychiatric disorders [131,132], these data further support lurasidone’s neuroprotective profile.

#### 3.3.3. Clinical Findings

Interestingly, a clinical study compared lurasidone with olanzapine in schizophrenic patients, assessing their ability to modulate serum neurotrophins. Both olanzapine and lurasidone significantly increased NGF and NT3 levels, with no statistically significant difference between the two treatment groups. However, serum BDNF levels were higher following treatment with olanzapine compared to lurasidone [133].

To sum up, it is evident that the data on the neurotrophic effects of lurasidone are more consistent than those of any other SGAs: in any case, they need to be further deepened.

### 3.4. Iloperidone

#### 3.4.1. Pharmacological Features and Clinical Context

Iloperidone is a novel antipsychotic primarily meant for the treatment of psychosis, based on its dopaminergic and serotonergic receptor antagonism. Specifically, it displays marked antagonism for D3, 5-HT2A and α1 receptors and also binds (with lower affinity) α2c, D2, D4, 5-HT1A, 5-HT2C, and 5-HT6 receptors. It exhibits negligible affinity for muscarinic μ1–μ5 receptors and for dopamine/norepinephrine reuptake transporters. Orthostatic hypotension is a principal adverse event, stemming from its high affinity for α1-adrenergic receptors, whereas the risk of anticholinergic side effects remains lower than with other antipsychotics [134,135]. Most existing research has focused on iloperidone’s efficacy in treating schizophrenia [123,136], although benefits have also been observed in mixed states of bipolar disorder [137].

#### 3.4.2. Potential Neurotrophic Relationship

At present, none of the literature directly correlates iloperidone with significant effects on known neurotrophic factors. Nevertheless, such a relationship is conceivable. Some scholars found a link between iloperidone administration and the gene encoding ciliary neurotrophic factor (CNTF) [138]. The CNTF belongs to the neuropoietic cytokine family, playing a key role in neuronal survival and differentiation [32,139] and has garnered clinical interest after reports of its possible association with schizophrenia [140]. Specifically, the presence of the null FS63TER allele of the rs1800169 polymorphism in patients homozygous for this mutation was linked to improved psychotic symptoms under iloperidone treatment, as measured by standard assessment scales. However, caution is warranted because the association between CNTF mutation and schizophrenia remains controversial; subsequent attempts to replicate the findings have been inconclusive [140,141,142]. Another distinctive property of iloperidone is its binding to α1 receptors, whose expression in the hippocampus may contribute to memory and long-term potentiation [143]. Thus, the increased hippocampal α1 receptor expression observed with iloperidone—a feature not shared by other APs—warrants further clinical research and indirectly suggests a possible involvement of this compound in neurotrophism [144].

### 3.5. Lumateperone (ITI-007)

#### 3.5.1. Pharmacological Profile and Clinical Use

Lumateperone is an antipsychotic of the butyrophenone class, approved in the United States by the FDA in late 2019 for the treatment of schizophrenia [145]. It is also under investigation for managing behavioral disorders related to neurodegenerative diseases, sleep disturbances, and mood disorders [146]. Lumateperone acts on dopaminergic, serotonergic, and glutamatergic systems [147], including robust antagonism at 5-HT2A, partial agonism at presynaptic and postsynaptic D2 receptors, the inhibition of 5-HT reuptake, and modulatory effects on D1 receptors. These properties may underlie potential antidepressant actions and improvement in negative symptoms of schizophrenia. Additionally, lumateperone enhances NMDA receptor function [148] and seems to have negligible affinity for 5-HT2C, H1, α-adrenergic, and muscarinic receptors—possibly minimizing certain adverse effects seen in other SGAs [149]. Clinical trials have shown its efficacy in schizophrenia [150,151,152].

#### 3.5.2. Neurotrophic Data

Currently, no data exist on lumateperone’s direct interaction with neurotrophism. Further research is warranted to elucidate any potential neuroprotective or neurotrophic benefits.

### 3.6. Pimavanserin

#### 3.6.1. Pharmacological Profile, Clinical Indications

Pimavanserin is a recently introduced antipsychotic approved for hallucinations and delusions in Parkinson’s disease (PD) psychosis [153]. It is also under investigation for similar symptoms in Alzheimer’s disease, agitation, and MDD, with phase II trials completed for adjunctive treatment of schizophrenia [154]. Unlike other SGAs, pimavanserin is an inverse agonist and antagonist at 5-HT2A receptors—and to a lesser extent 5-HT2C receptors—exhibiting no significant affinity for dopamine, histamine, muscarinic, or adrenergic receptors. Because 5-HT2A receptors are abundant in hippocampus, amygdala, nucleus accumbens, striatum, hypothalamus, and cortex, pimavanserin’s mechanism essentially reverses 5-HT2A activity when no agonist is present and blocks agonist-driven activity; this contrasts with the agonist action of hallucinogens such as LSD [153].

#### 3.6.2. Animal/Translational Evidence

Given that antipsychotic compounds with neuroprotective properties tend to have higher affinity for 5-HT2A versus D2 receptors, pimavanserin appears to be a promising candidate for novel antipsychotic development with a neurotrophic profile. Though data remain preliminary, recent evidence shows that pimavanserin, along with another 5-HT2A inverse agonist M100907, exerts protective effects against 1-methyl-4-phenylpyridinium (MPP+)-induced cell death in dopaminergic neurons. In the case of pimavanserin, such neuroprotective actions were linked to the increased release of BDNFs and glial cell line-derived neurotrophic factors (GDNFs) [155]. Notably, these neuroprotective effects were blocked by anti-GDNF antibodies but not by antibodies against the tyrosine receptor kinase B, suggesting a GDNF-dependent mechanism. This finding is consistent with a previous study reporting increased GDNF levels in patients with schizophrenia following SGA treatment [156].

In conclusion, if corroborated by clinical data in psychotic patients, preliminary and preclinical data would suggest that pimavanserin might promote neurotrophic processes.

### 3.7. Roluperidone (MIN-101)

#### 3.7.1. Pharmacological Profile and Clinical Considerations

Roluperidone, also known as MIN-101, is among the latest antipsychotic studied for potential utility in schizophrenia treatment. It binds with high affinity to sigma-2 and 5-HT2A receptors, as well as α1-adrenergic receptors, while displaying lower or negligible affinity for dopamine, muscarinic, and histamine receptors [157,158]. Despite the apparent lack of direct action on dopamine receptors, an indirect modulatory effect is still plausible since sigma-2 receptors have been implicated in regulating both dopamine [159,160] and glutamate [161]. Reported adverse effects include headache, insomnia, nausea, and asthenia, whereas body weight and metabolic parameters seem to remain largely unaffected [158]. Additionally, roluperidone may improve cognitive functions in patients with schizophrenia [157].

#### 3.7.2. Preclinical Neurotrophic Findings

So far, data regarding any neuroprotective effects of roluperidone remain sparse, as most efforts focus on demonstrating its clinical efficacy. Nevertheless, a preclinical study revealed a dose-dependent increase of about 20% in BDNF levels in hippocampal neurons after three days of roluperidone use [162]. This effect was comparable to that observed with pridopine, a sigma-1 receptor agonist mostly investigated in Huntington’s disease research [163].

**Table 1:** Receptor affinity profiles and neurotrophic effects.

Drug	D2	D3	5-HT1A	5-HT2A	5-HT2B	5-HT2C	5-HT7	α1 Adrenergic	α2C Adrenergic	H1	M1	Sigma-2	NMDA	Neurotrophic Implications
Brexpiprazole	+/− (D2) (Reduced intrinsic activity)	Not specified	+/−	−++ (Potent)	Not specified	Not specified	Not specified	−++ (α1b)	−++ (α2c)	+ (Moderate)	0	0	0	- Enhances neurite outgrowth via 5-HT1A and 5-HT2A receptors [100] - Involves Ca^2+^ signaling and Hsp90 - Increases BDNF levels when combined with fluoxetine [104,105] - Improves dendritic spine density
Cariprazine	+/−	+/− +++ (Higher affinity for D3)	+/−	0	−	+	0	0	0	0	0	0	0	- May increase D3 receptors mediated by BDNF [114] - Potential antidepressant activity via neuroadaptive changes [113] - Protective effect on cognitive functions [112]
Lurasidone	−++ (Full antagonist)	0	+/−	−++ (Full antagonist)	0	+	−++	0	−+	0	0	0	0	- Modulates BDNF expression in prefrontal cortex and hippocampus [126] - Affects synaptic proteins GFAP and PSD-95 - Influences mTOR and EEF2 pathways [129] - Potential cognitive benefits in schizophrenia
Iloperidone	−+	−++	−+	−++	0	−+	0	−++	−+	0	0	0	0	- Possible link with CNTF gene affecting neuronal survival [138] - α1 antagonism in hippocampus may influence memory and LTP [144] - Indirect involvement in neuroplasticity
Pimavanserin	0	0	0	−++ (Inverse agonist)	−++ (Inverse agonist)	0	0	0	0	0	0	0	0	- Neuroprotective effects in dopaminergic neurons via GDNF [155] - Increases BDNF and GDNF release - Potential benefits in Parkinson’s disease psychosis
Roluperidone	0	0	0	−++	0	0	0	−+	0	0	0	++ (High affinity)	0	- Increases BDNF levels in hippocampal neurons [162] - Potential neurotrophic effects via sigma-2 receptor modulation - May improve cognitive functions in schizophrenia [157]
Lumateperone	+/− (Presynaptic D2) (Partial agonist)	0	0	−++	0	0	0	0	0	0	0	0	++ (Modulator)	- Modulates NMDA glutamatergic receptors [148] - Enhances serotonergic neurotransmission - No direct evidence yet of neurotrophic factor modulation [152] - Potential area for future research

+/−: Partial agonist; −: Antagonist; −+, −++: Antagonist with moderate to high affinity; +, ++, +++: Agonist with low to high affinity; 0: Negligible or no affinity; D2, D3: Dopamine receptor subtype 2 and 3; 5-HT1A, 5-HT2A, etc.: Serotonin receptor subtypes; α1, α2C: Alpha adrenergic receptor subtypes; H1: Histamine receptor subtype 1; M1: Muscarinic acetylcholine receptor subtype 1; Sigma-2: Sigma receptor subtype 2; and NMDA: N-Methyl-D-aspartate glutamate receptor.

Notes on Neurotrophic Implications:

- Brexpiprazole: Enhances neurite outgrowth and increases BDNF levels, suggesting promotion of neuroplasticity.
- Cariprazine: May modulate BDNF-mediated neuroadaptive changes due to high affinity for D3 receptors.
- Lurasidone: Increases BDNF expression and affects proteins involved in synaptic plasticity, potentially improving cognitive deficits.
- Iloperidone: Indirect evidence suggests involvement in neuroplasticity through α1 receptor antagonism.
- Pimavanserin: Neuroprotective via GDNF release, beneficial in dopaminergic neuron survival.
- Roluperidone: Increases BDNF levels; sigma-2 receptor affinity may contribute to neurotrophic effects.
- Lumateperone: Modulates glutamatergic neurotransmission; potential neuroplastic benefits need further investigation.

**Table 2:** Preclinical (animal/in vitro) studies suggesting neurotrophic or neuroprotective effects.

Drug	Reference	Experimental Model	Key Neurotrophic Findings
Brexpiprazole	Ishima et al. [100] Ma et al. [104,105]	- P12 cell line (NGF-induced neurite outgrowth) - Mouse models of inflammation-induced depression and social defeat stress	- Neurite outgrowth enhanced via 5-HT1A/5-HT2A receptors, Ca2+ (IP3) signaling - Combination with fluoxetine reduced immobility and increased BDNF levels in PFC, hippocampus, and reversed spine density loss in rodents
Cariprazine	Papp et al. [111] Zimnisky et al. [112] Leggio et al. [113] Choi/Gyertyán/Sokoloff/Kiss [114,115,116,117]	- Rat chronic mild stress (anhedonia) - Mouse model (cognitive/PCP-induced deficits) - D3 receptor expression assays	- Reduced anhedonia-like behavior in CMS - Putative neuroadaptive changes linked to D3 receptor partial agonism - BDNF implicated in D3 receptor expression/upregulation following chronic cariprazine
Lurasidone	Fumagalli et al. [126] Calabrese et al. [128] Luoni et al. [129]	- Rat stress/depression models (PFC, hippocampus) - Lurasidone + valproate co-administration	- Increased BDNF mRNA/protein in PFC and hippocampus - Neuroadaptive changes in ventral hippocampus (synergy with valproate) - Modulation of GFAP, PSD-95, mTOR, EEF2 involved in synaptic plasticity
Iloperidone	Szot et al. [143] Choi and Tarazi [144]	- Potential α1 receptor expression in hippocampus - Long-term receptor binding assays	- Indirect neurotrophic relevance: α1 receptor upregulation in hippocampus may influence memory/LTP (the study of [144] shows changes in serotonin and adrenoceptor subtypes). No direct BDNF/NGF measurement, but points to possible neuroplastic role
Lumateperone	(No direct preclinical data on neurotrophism)	- Not applicable	- No current evidence for neurotrophic outcomes in animals/in vitro
Pimavanserin	Lavigne et al. [155]	- Primary dopaminergic neurons exposed to MPP+	- Protective against MPP^+^-induced cell death - Increased BDNF and especially GDNF release (blocked by anti-GDNF antibodies), indicating GDNF-dependent neuroprotection
Roluperidone	Minerva Neurosciences [162]	- In vitro hippocampal neuron culture	- ~20% increase in BDNF after 3 days of treatment - Comparable to pridopine (a sigma-1 agonist) in BDNF induction

**Abbreviations:** NGF = nerve growth factor; PFC = prefrontal cortex; CMS = chronic mild stress; PCP = phencyclidine; GFAP = glial fibrillary acidic protein; PSD-95 = postsynaptic density protein 95; mTOR = mammalian target of rapamycin; EEF2 = eukaryotic elongation factor 2; and GDNF = glial cell line-derived neurotrophic factor.

**Table 3:** Clinical (human) findings on neurotrophic effects.

Drug	Reference	Study Population/Design	Neurotrophic-Related Observations
Brexpiprazole	(Various acute/chronic trials in schizophrenia, MDD, bipolar depression) [92,93,94,95,96,97,98,99]	- Multiple RCTs and open-label studies focusing on efficacy, tolerability, and mood - No direct BDNF/NGF measurement in humans reported	- Demonstrated efficacy in schizophrenia (acute and relapse prevention) and adjunctive MDD - No specific biomarkers of neurotrophism examined in these clinical trials
Cariprazine	(Adjunctive in MDD) [109,110] (Schizophrenia trials) [106,107]	- RCTs assessing symptom improvement and tolerability - No direct measurement of BDNF/other trophic factors	- Efficacy in schizophrenia and bipolar I disorder; potential cognition benefits - No published neurotrophic biomarkers in clinical settings
Lurasidone	Jena et al. (2019) [127]	- Randomized controlled trial comparing lurasidone vs. olanzapine in unmedicated schizophrenia	- Increased NGF and NT3 in both treatment arms - BDNF significantly higher with olanzapine vs. lurasidone - Only direct human trial focusing on serum neurotrophins
Iloperidone	Lavedan et al. [138]	- Genetic polymorphism study (ciliary neurotrophic factor, CNTF) in schizophrenic patients - Sub-analysis of an iloperidone clinical trial	- Presence of CNTF polymorphism (FS63TER allele) linked to better symptom improvement with iloperidone - Suggests an indirect association of CNTF genotype and clinical response
Lumateperone	(Clinical trials in schizophrenia) [150,151,152]	- Phase 2/3 RCTs demonstrating antipsychotic efficacy and safety - No neurotrophic biomarker data published	- Efficacy in symptoms control but no direct measurement of BDNF/NGF or other markers
Pimavanserin	(Parkinson’s disease psychosis; dementia-related psychosis; MDD) [153,154]	- Trials primarily evaluating safety and efficacy in PD psychosis, MDD adjunct - No data on BDNF/NGF in humans	- Early-phase studies for schizophrenia as adjunct - Neuroprotective potential inferred from preclinical data, no direct clinical neurotrophic measure
Roluperidone	(Schizophrenia negative symptoms) [157,158]	- RCTs primarily focusing on symptom reduction and safety - No direct BDNF measurement in patients	- Preliminary data suggest improvement in negative symptoms, cognition - No human neurotrophic biomarker data reported

**Abbreviations:** BDNF = brain-derived neurotrophic factor; NGF = nerve growth factor; NT3 = neurotrophin-3; CNTF = ciliary neurotrophic factor; PD = Parkinson’s disease; MDD = major depressive disorder; and RCT = randomized controlled trial.

## 4. Discussion

The evolving body of literature increasingly highlights the potential of third-generation antipsychotics (TGAs) to exert more pronounced influences on neuroplasticity and neurotrophic factors compared to earlier classes of antipsychotics. Although second-generation antipsychotics (SGAs) have been in use for over two decades and are known for going beyond D2 receptor antagonism—exemplified by the SGA lurasidone, which targets 5-HT7 receptors and has been linked to increases in BDNF under stress [126]—their real-world neuroprotective or pro-cognitive benefits remain limited. TGAs, on the other hand, leverage distinct pharmacological profiles, often characterized by partial agonism at D2/D3 receptors or more nuanced serotonin–dopamine modulation, thereby refining the therapeutic targeting of neuroplastic processes. Neuroplasticity, defined as the brain’s capacity to reorganize itself by forming new neuronal connections, relies on pivotal neurotrophic factors such as BDNFs and NGFs, which foster neuronal health, synaptic growth, and tissue repair. While SGAs like lurasidone provide a useful template for broad receptor activity, the pharmacological diversity of TGAs—particularly in their dopaminergic and serotonergic receptor interactions—may further heighten their impact on these pathways. In addition to antagonism at D2 receptors, TGAs frequently incorporate more potent or selective actions at 5-HT1A and 5-HT2A receptors, potentially delivering more robust neuroprotective or pro-cognitive outcomes than seen with older compounds. This enhanced receptor interplay could offer more sustained synaptic adaptation, thereby strengthening the rationale for TGAs as a meaningful step beyond the foundational but sometimes inconsistent neuroplastic effects observed with SGAs (Figure 1).

Brexpiprazole and cariprazine both act on dopaminergic receptors but differ in their affinities and intrinsic activities. Brexpiprazole is a partial agonist at D2 receptors with reduced intrinsic activity, which may decrease side effects like restlessness and akathisia [90]. It also shows moderate affinity for histamine H1 receptors, contributing to a lower risk of weight gain and sedation [89]. Cariprazine, conversely, exhibits a higher affinity for D3 receptors over D2 receptors, acting as a partial agonist at both sites [106,107]. The preferential targeting of D3 receptors is significant, as these receptors are implicated in cognitive functions and mood regulation. It has been hypothesized that enhanced D3 activity might possibly lead to neuroadaptive changes associated with antidepressant effects [113]. Furthermore, cariprazine’s ability to increase D3 receptor levels may be mediated by the BDNF, which controls the development and expression of D3 receptors [114,116], therefore suggesting that cariprazine’s dopaminergic profile might positively influence neurotrophic processes.

Brexpiprazole, different from cariprazine, seems to enhance neurite outgrowth in PC12 cells via 5-HT1A and 5-HT2A receptors, involving calcium signaling through IP3 receptors and the molecular chaperone Hsp90 [100]. Additionally, brexpiprazole combined with fluoxetine showed antidepressant effects in mice, attenuating decreases in BDNF levels and improving dendritic spine density in brain regions associated with mood regulation [104,105].

Lurasidone acts as a full antagonist at 5-HT2A receptors and a partial agonist at 5-HT1A receptors, with marked affinity for 5-HT7 receptors [121,122], therefore possibly contributing to cognitive enhancement and memory processes [123]. Lurasidone has been shown to modulate BDNF expression at both mRNA and protein levels in the prefrontal cortex and hippocampus, suggesting a neuroprotective role that could ameliorate cognitive deficits in schizophrenia [126]. It might also affect synaptic and neuroplastic proteins, such as GFAP and PSD-95, and signaling pathways such as mTOR and EEF2, which are crucial for synaptic plasticity and memory consolidation [129].

Iloperidone shows high antagonistic affinity for 5-HT2A receptors [134] and, although direct evidence linking iloperidone to neurotrophic effects remains limited, its serotonergic profile suggests potential involvement. Furthermore, a study found a correlation between iloperidone treatment and the CNTF, which plays a role in neuronal survival and differentiation [138]; however, this association requires further validation due to inconsistent replication.

Pimavanserin, an inverse agonist and antagonist at 5-HT2A and 5-HT2C receptors, with no significant dopaminergic activity [153], might be endowed of a neuroprotective role in dopaminergic neurons, potentially by the release of GDNFs [155].

Lumateperone also antagonizes 5-HT2A receptors and inhibits serotonin reuptake, enhancing serotonergic neurotransmission [147]. Additionally, it modulates glutamatergic NMDA receptors [148]. While these properties suggest potential neurotrophic effects, the current literature lacks direct evidence linking lumateperone to neurotrophic modulation.

Brexpiprazole’s antagonism at the level of α1b/2c adrenoreceptors [90] may contribute to its neurotrophic effects, as adrenergic receptors are involved in neural plasticity. Iloperidone’s high affinity for α1 receptors [135] is notable; α1 receptors in the hippocampus are implicated in memory and long-term potentiation [143]. Iloperidone-induced increases in α1 receptors in the hippocampus may influence neuroplastic processes [144].

Lurasidone’s moderate antagonism at α2C receptors [122] could also play a role in cognitive functions and neuroplasticity. Its low affinity for histamine H1 and muscarinic M1 receptors reduces risks of sedation and weight gain [124], potentially improving treatment adherence and indirectly supporting neuroplasticity through sustained therapy.

Roluperidone exhibits high affinity for sigma-2 receptors and 5-HT2A receptors and acts on α1-adrenergic receptors, with negligible affinity for dopaminergic receptors [157,158]. Sigma-2 receptors are implicated in the modulation of neurotransmitter systems, including dopamine and glutamate [160,161]. Preliminary data suggest that roluperidone increases BDNF levels in hippocampal neurons indicating potential neurotrophic effects via sigma receptor pathways [162].

Lumateperone’s modulation of NMDA glutamatergic receptors sets it apart from other SGAs [148]. The NMDA receptors are crucial for synaptic plasticity and neuroplastic processes. Although direct evidence of lumateperone’s impact on neurotrophic factors is not yet available, its glutamatergic activity suggests potential neuroplastic benefits.

Advancements in biomarker research and neuroimaging techniques hold the potential to assess individual neurotrophic response profiles. By measuring baseline levels of neurotrophic factors like BDNFs and monitoring changes during treatment, clinicians may better predict therapeutic responses and tailor interventions accordingly [164]. For example, patients with low baseline BDNF levels might derive greater benefit from SGAs known to enhance BDNF expression, such as lurasidone or brexpiprazole [100,126]. Pharmacogenetic testing could further identify genetic variations affecting neurotrophic factor expression or receptor sensitivity, guiding personalized medication selection and dosing strategies.

An additional area warranting attention is the potential therapeutic role of TGAs in substance-induced psychoses, particularly those related to cannabis use. Evidence suggests that cannabis exposure may interfere with neurotrophin synthesis and function—notably NGFs, BDNFs, and GDNFs—thereby contributing to the neurotoxic processes implicated in psychosis [165]. By targeting both dopaminergic and serotonergic pathways, and in some cases upregulating neurotrophic factors, TGAs could theoretically counteract the deleterious impact of psychostimulants on neuronal plasticity. While research specifically exploring TGAs in cannabis-related psychosis remains sparse, the mechanistic rationale underscores an important therapeutic opportunity. Clarifying whether TGAs indeed mitigate the neurochemical disruptions induced by Δ-9-THC or enhance neuronal resilience in vulnerable individuals represents a promising direction for future clinical trials.

## 5. Limitations

Despite these promising findings, several limitations persist in the current body of research. The majority of available studies are preclinical or involve relatively small clinical samples, limiting the generalizability of the results. Moreover, substantial variability in experimental designs and biomarker assessments complicates direct comparisons between different TGAs. It is also important to acknowledge that neuroplastic effects observed in animal models may not necessarily translate into analogous benefits in human populations. Large-scale, well-controlled clinical studies are needed to further elucidate the potential neuroprotective properties of these compounds. In addition, our own review has certain constraints. First, we adopted a scoping-type methodology rather than a fully systematic approach, which did not include a formal risk-of-bias appraisal or meta-analysis. This broader perspective may have introduced variability in the studies included. Second, we focused primarily on English-language articles, potentially overlooking relevant findings in non-English literature. Third, while we grouped newer agents under the “TGA” umbrella, there is no universally accepted definition of TGAs, and some agents (e.g., iloperidone or pimavanserin) may be variously categorized in the literature. Future research should address these methodological gaps and classification inconsistencies to provide a more comprehensive understanding of TGAs’ neuroprotective and neurotrophic effects.

## 6. Conclusions

Emerging evidence suggests that third-generation antipsychotics (TGAs) may confer distinct advantages over their predecessors, particularly in the modulation of neuroplasticity and neuroprotection. Recent studies indicate that compounds such as cariprazine and brexpiprazole, through their partial agonism at dopamine D_2_ and D_3_ receptors, not only mitigate psychotic symptoms but may also enhance neuroplastic processes. These effects could potentially translate into cognitive improvements and greater neuronal resilience in patients with psychotic disorders. However, while the neurobiological rationale for these effects is compelling, robust clinical validation remains necessary.

## 7. Recommendations for Future Research

A deeper understanding of the neurotrophic and neuroplasticity-enhancing properties of TGAs requires further investigation through large-scale, long-term studies capable of monitoring patients over extended periods, ideally spanning several years. Such longitudinal research would provide crucial insights into the sustained effects of TGAs on neuroplastic mechanisms and their implications for disease progression and functional outcomes in psychiatric disorders such as schizophrenia and mood disorders. To achieve this, it is imperative to refine methodological approaches by establishing standardized protocols for measuring neurotrophic biomarkers, including brain-derived neurotrophic factors (BDNFs) and other neuroplasticity-related factors. Ensuring homogeneity in sample collection, processing, and analysis across studies would greatly enhance the comparability and reliability of findings, addressing one of the main challenges in this field. Furthermore, the integration of advanced neuroimaging techniques and biomarker-based assessments may significantly contribute to elucidating the structural and functional changes associated with TGAs’ modulation of neurotrophic pathways. Beyond methodological improvements, a paradigm shift in clinical research strategies is necessary to fully explore the therapeutic potential of TGAs. Strengthening collaborations between territorial healthcare services and academic institutions could be pivotal in this regard. Given that territorial healthcare providers manage the majority of patients with chronic psychiatric conditions over long periods, they possess a wealth of longitudinal clinical data that, in most cases, remains underutilized. By fostering partnerships with universities and research institutions, which offer advanced methodological expertise and scientific resources, it would be possible to design and implement retrospective and prospective large-scale studies, integrating real-world clinical data with rigorous scientific methodologies. This approach would not only improve the external validity of research findings but also facilitate a more comprehensive evaluation of TGAs’ effectiveness in everyday clinical practice. Ultimately, such an integrative research framework could lead to more personalized treatment strategies, improving both symptom management and long-term neuroprotection in psychiatric patients. By advancing our understanding of how TGAs influence neuroplasticity, these efforts may pave the way for a redefinition of treatment paradigms, shifting the focus from symptom control alone to a broader strategy aimed at enhancing neuronal resilience and functional recovery.

## Abbreviations

The following abbreviations are used in this manuscript:

APs	Antipsychotic drugs
SGFs	Second-generation antipsychotics
TGFs	Third-generation antipsychotics
BDNF	Brain-derived neurotrophic factor
FGAs	First-generation antipsychotics
SGAs	Second-generation antipsychotics
EPSs	Extrapyramidal symptoms
FDA	Food and Drug Administration
MDD	Major depressive disorder
GFs	Growth factors
GDNF	Glial cell line-derived neurotrophic factor
NT-3	Neurotrophin 3
NT-4	Neurotrophin 4
TrkA, TrkB, TrkC	Tropomyosin receptor kinase A, B, C
p75NTR	p75 neurotrophin receptor
NGF	Nerve growth factor
PSD	Postsynaptic density
IEG	Immediate early gene
PFC	Prefrontal cortex
fMRI	Functional magnetic resonance imaging
NET	Norepinephrine transporter
Hsp90	Heat shock protein 90
IP3	Inositol 1,4,5-triphosphate
DG	Dentate gyrus
5-HT2A	Serotonin receptor 2A
α2C	Alpha-2C Adrenergic Receptor
5-HT7	Serotonin receptor 7
M1	Muscarinic acetylcholine receptor M1
H1	Histamine receptor 1
α1A	Alpha-1A Adrenergic Receptor
α2A	Alpha-2A Adrenergic Receptor
Gfap	Glial fibrillary acidic protein
Psd95	Postsynaptic density protein 95
mTOR	Mammalian target of rapamycin
EEF2	Eukaryotic elongation factor 2
PD	Parkinson’s disease
